# Animal Movements and FMDV Transmission during the High-Risk Period of the 2001 FMD Epidemic in Uruguay

**DOI:** 10.1155/2023/8883502

**Published:** 2023-11-11

**Authors:** María V. Iriarte, José L. Gonzáles, Andrés D. Gil, Mart C. M. de Jong

**Affiliations:** ^1^Quantitative Veterinary Epidemiology, Wageningen University and Research, Wageningen, Netherlands; ^2^Department of Epidemiology, Bioinformatics and Animal Models, Wageningen Bioveterinary Research, Lelystad, Netherlands; ^3^Unidad de Epidemiología, Dirección General de Servicios Ganaderos, Ministerio de Ganadería Agricultura y Pesca, Montevideo, Uruguay; ^4^Facultad de Veterinaria, Universidad de la República de Uruguay, Montevideo, Uruguay

## Abstract

During the 2001 foot-and-mouth disease (FMD) epidemic in Uruguay, many farms were already infected and foot-and-mouth disease virus (FMDV) had spread throughout the country by the time the first outbreak was detected and a ban on animal movements was implemented. Before this ban, movements of infected animals between livestock premises were probably one of the main factors contributing to the spread of the disease. Understanding and quantifying this contribution allow identifying risk premises or risk areas to help policymakers to implement effective interventions and enhance targeted surveillance. The aim of this study was to describe, visualize, and analyze the network of livestock movements between livestock premises during the initial phase of the 2001 FMD epidemic in Uruguay and gain insight into the risk of transmission by estimating the between herd basic reproduction number (*R*_H_) before a ban on animal movements was implemented. Here, we derived *R*_H_ from the average number of outcontacts of infected premises and the average probability that a contact leads to infection. Additionally, we analyzed the current (2022) network of livestock movements in Uruguay, for the same period as in 2001, and estimated *R*_H_ assuming the same probability of infection as in 2001. We found that the movements of infected animals during the high-risk period of this epidemic—i.e., the period between FMDV introduction and the detection of the index case—had an important contribution to the virus spread among premises (*R*_H_ = 1.48). Livestock markets and highly connected farms were responsible for the early long-distance spread of FMDV. The analysis of the 2022 network shows that this network is similar to that of 2001 and highlights the importance of targeting highly connected premises, particularly livestock markets, for surveillance, target early detection, and implement interventions during epidemics.

## 1. Introduction

Foot-and-mouth disease (FMD) is a contagious viral disease that affects cloven-hoofed animals. Susceptible animals can be infected by the direct contact with an infectious animal or by indirect contact via a contaminated environment [1–3]. The main route of transmission between farms is by the movement of infected animals. Foot-and-mouth disease virus (FMDV) also can be spread by contaminated animal products (particularly important in pigs) and by fomites such as transport vehicles, farming tools, milking machines, etc. [4, 5].

In April 2001, a FMD major epidemic affected Uruguay. A previous study showed that during the high-risk period (HRP)—i.e., the period between FMDV introduction and the detection of the index case—of this epidemic, probably more than 200 farms were already infected [6]. During the HRP, between farms transmission could have taken place through movement of animals or contaminated fomites since control measures had not yet been implemented and farming activities remained unchanged.

The movement of infected animals between farms is probably one of the main factors contributing to the spread of diseases before interventions are implemented. For example, the long-distance movement of infected sheep to markets before FMDV was first diagnosed in the UK in 2001 has been reported as a determinant of the size of the epidemic, highlighting the role that livestock markets and dealers played in FMDV early spread [7].

Livestock movements can be analyzed by using network analysis and graph theory, providing valuable information on the contact structure among livestock premises, on which infectious diseases can spread [8, 9]. Knowledge of this structure allows disease dynamics modeling, which is an important tool to interpret epidemic data and to predict future scenarios to inform policymaking [10, 11].

Network analysis in veterinary epidemiology has been performed to assess the risk of disease spread through livestock movements and the identification of targets for surveillance and interventions [12–14]. Application of network analysis to data from epidemics can be used to gain insights in the role of animal movements in the spread of infection [15, 16].

In Uruguay, a livestock stock and movement control system were established by law in 1973. Therefore, the movements of animals between farms have been recorded since many years before 2001. The availability of these data has been used by the Veterinary Services as a crucial piece of information during outbreaks investigations to trace potentially infected animals. More recently, the data of cattle movements from 2008 to 2013 were also used by researchers to analyze its patterns [17]. This study provided valuable information to identify risk farms for infection and spread of infectious diseases and to guide the development of targeted surveillance and control interventions. However, the analysis of livestock movements during the 2001 FMD epidemic in Uruguay has not yet been performed. The analysis of this data provides the opportunity to better understand specifically the role of animal movements in the transmission of FMDV and, thus, contribute to the development and parameterization of epidemiological models of its spread.

The aim of this study is to describe, visualize, and analyze the network of livestock movements between livestock premises during the initial phase of the 2001 FMD epidemic in Uruguay and gain insight into the risk of transmission by estimating the between herd basic reproduction number (*R*_H_) before a ban on animal movements was implemented. For this analysis, *R*_H_ is the average number of new infected farms that were caused by a typical infected farm through the movements of infected animals. This study will contribute to the parameterization of FMDV spread models that consider the contact structure among premises through animal movements, and it will provide a better understanding of the role of livestock networks during the initial phase of an epidemic.

## 2. Materials and Methods

### 2.1. Data Source

The Livestock Control Office (DICOSE) under the Ministry of Livestock, Agriculture and Fishery (MGAP) manages the livestock stock and movements control system of Uruguay, which was established by law in 1973. In short, this system keeps a register of livestock owners, by assigning them a unique registration number, and who are then obliged to update their livestock stock record annually. In addition, there is an ownership and movement form for registration of livestock movements. This form contains information about livestock ownership, type of operation, the source and the recipient farm, the livestock species moved, the number of animals, the animal identification, the means of transport used, the places where the cattle will be moved from and to, who is responsible for moving the animals, and when the movement will be done. The movements can be audited at anytime and anywhere in the national territory [18].

In addition to the animal movements database, we used the database gathered by the Veterinary Services of Uruguay during the 2001 FMD epidemic to determine which farm was infected with FMDV during the HRP. These official reports were merged with the data on the location and livestock composition for all Uruguayan farms that kept cattle or sheep in 2001 (DICOSE Annual Affidavit, 2001). The 2001 data of pig-only farms were not available.

### 2.2. Study Population

In Uruguay, the farming of cattle and sheep is distributed throughout the country, while pigs are less relevant in number of animals and farms. In 2001, the stocks of cattle, sheep, and pigs were around 10.7 million heads of cattle kept in 43,724 farms, 12 million heads of sheep kept in 25,594 farms, and almost 100,000 pigs kept in 3,461 farms (DICOSE Annual Affidavit, 2001). For this study, farms that raised cattle were classified regarding its production purpose as beef (38,872), dairy (3,194), and mixed (dairy and beef) (1,658) farms. In addition, farms were categorized into four herd size categories from the quartiles (<36 heads, 36–134 heads, 134–463 heads, and >463 heads).

For the purpose of this study, we considered the movements of live cattle and sheep during the initial phase of the 2001 FMD epidemic in Uruguay. The initial phase of this epidemic refers to the period between the estimated date of FMDV introduction (April 9, 2001) and the enforcement of animal movements restrictions (April 27, 2001), which occurred 3 days after the detection of the index case. Based on a previous study [6], the population consisted of 44,693 uninfected farms and 242 farms that were infected during the HRP.

### 2.3. Analysis

#### 2.3.1. Description of Livestock Movements' Network during the Initial Phase of the Epidemic

First, a general directed network of cattle and sheep movements was constructed for the initial phase of the epidemic, which was named the general network (GN). Each node represented a livestock operation (farm, livestock market, or slaughterhouse) listed as a source or recipient in the animal movement database. The directed link between nodes represented the movement of livestock from source to recipient livestock operations on a specific date. Then, another network was developed after removing slaughterhouses as they were considered end points of FMDV transmission, which was named the general network without slaughterhouses (GNWS). Finally, we defined a network called the transmission network (TN), which included all links from or to farms that were infected before the ban of animal movements was enforced. The geographical distances of livestock movements between premises were analyzed for all the networks.

A more in-depth network analysis, stratified by production purpose and herd size, was limited due to the low number of movements involving dairy farms in the TN and missing of demographic data for approximately 7% of farms that moved animals during the study period. Therefore, we only computed basic summary statistics of animal movements for these strata.

The connectivity of each farm was measured by its indegree and outdegree. In this study, the indegree represented the number of contacts that provide animals to a specific livestock holding (i.e., inward livestock movements). On the other hand, the outdegree was the number of contacts that received animals from a specific livestock holding (i.e., outward livestock movements). We further measured the betweenness for each premise of the GNWS. The betweenness measures how frequently a vertex lies on the shortest paths between any two vertices in the network [19].

In addition, other metrics were estimated to assess the level of connectedness of the GNWS. We estimated the average path length, which refers to the average shortest paths (number of steps) among reachable pairs of nodes in the network. This metric was compared to the average path length of 1,000 random networks of same size and density as our network generated by using the algorithm developed by Erdös and Renyi [20]. We also estimated the number of nodes that can be reached in two steps from each node of the network.

We used the igraph package in R Statistical Software (version 4.2.2) for these analyses [21].

#### 2.3.2. Estimation of the Basic Reproduction Number (R_H_)

The basic reproduction number (*R*_0_) is the average number of secondary infections caused by a typical infectious individual in a population where all other individuals are susceptible [22]. The infection process implies that susceptible individuals must have contact with an infectious individual or with infectious material of infectious individuals. In our study, we considered herds (premises) as the units of analysis. For the estimation of *R*_H_, we assumed that susceptible premises only had contact with an infectious premise through the movements of infected animals. By definition, any infected premise through animal movements has been first infected by receiving infected animals itself before transmitting infection to other susceptible farms. This means that such a farm had at least one inward and one outward movements [23] and its distribution of outward movements is not the distribution of outward movements in the whole population. Here, we derived the reproduction number from the average number of outcontacts of infected premises ($\overset{―}{y}\prime$) and the probability that a contact leads to infection ($\overset{―}{q}$) based on the approach by Diekmann et al. [23]. For the derivation details, please refer to the appendix.

The average number of outcontacts of infected premises using that approach is given as follows:

(1) $$\begin{array}{c} \overset{―}{y}\prime =\frac{\text{cov}\left(x,y\right)}{\overset{―}{x}}+\overset{―}{y}, \end{array}$$

where *x* is the indegree and *y* is the outdegree variable with the observed covariance (cov(*x*, *y*)) and averages ($\overset{―}{x}$ and $\overset{―}{y}$).

The probability that a contact leads to an infection ($\overset{―}{q}$) was estimated from the number of farms that got infected before the movements restrictions were implemented divided by the total number of farms included in the TN described above.

The expected number of secondary cases per primary case in the initial phase of the epidemic equals:

(2) $$\begin{array}{c} R_{H}=\overset{―}{q}\left(\frac{\text{cov}\left(x,y\right)}{\overset{―}{x}}+\overset{―}{y}\right). \end{array}$$

Due to the possible uncertainty around the date of infection of each farm, we also estimated *R*_H_ assuming that the virus was introduced 1 week earlier.

The mean of in- and outdegree and covariance between them were estimated for livestock premises in the three networks of livestock movements in 2001 (GN, GNWS, and TN). Additionally, we estimated these metrics for the current network of livestock movements in Uruguay for the period between April 9, 2022 and April 27, 2022, which corresponds to the same period as the HRP of the 2001 FMDV epidemic. *R*_H_ was also estimated for current networks assuming the same probability of infection as that observed during the HRP.

## 3. Results

### 3.1. Description of Animal Movements during the Initial Phase of the Epidemic

**Figure 1:**
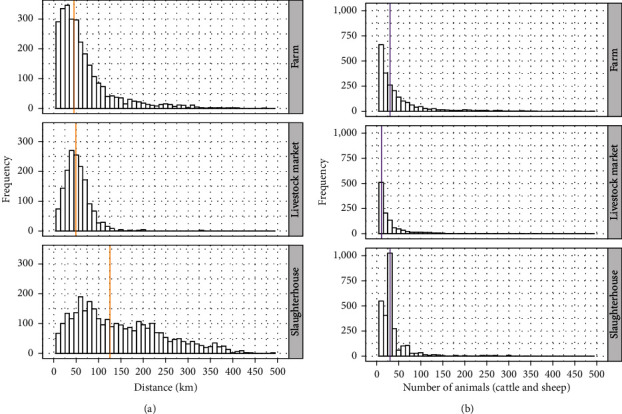
(a) Distance (km) distribution of livestock movements from farms split by the type of recipient premise (farm, livestock market, and slaughterhouse). (b) Distribution of the number of animals of livestock movements from farms split by the type of recipient premise. The vertical lines show the median of the distance and number of animals, respectively.

The GN, containing all cattle and sheep movements during the initial phase of the epidemic, consisted of 6,535 nodes and 7,695 links among them, with an average of 1.2 links per node and a median of 22 animals (cattle and sheep) and a median distance of 64.9 km per link. Most of the movements involved cattle only (86%), while 11% of them were sheep only and 3% included both species. Of the total movements from farms, 48.7% were to slaughterhouses with a median of 30 animals and a median distance of 125 km, 26.5% to livestock markets with a median of 11 animals and a median distance of 49.3 km, and 24.8% to other farms with a median of 30 animals and a median distance of 45 km (Figure 1).

From the total of farms that acted as source of animals, 89% of them corresponded to beef farms, 5.5% to dairy farms, and 5.5% to mixed farms. With respect to farm size, these farms were mostly (56.2%) large farms (>463 animals). Additionally, out of the total of farms that acted as recipients of animals, 90.6% comprised beef farms, 4.6% dairy farms, 4.5% mixed farms, and mostly (49.7%) large farms (Table S1).

Regarding the overall number of livestock premises that had recorded movements during the initial phase of the epidemic, 74 were infected and 6,456 were uninfected during the HRP.

The GNWS consisted of 4,590 nodes and 4,505 links with an average of 1.02 links per node and a median of 16 animals and a median distance of 48.3 km per link. Similarly to the GN, 81% of the movements involved cattle only, while 15% of them were sheep only and 4% were mixed movements (cattle and sheep). Of the total of movement from farms, 47.9% were movements between farms and 52.1% from farms to livestock markets. Regarding the production purpose and the farm size, the proportion of farms of different production types and herd sizes were similar to its proportion in the GN (Table S1). Figure 1 shows that the movements of animals between farms and from farms to livestock markets mostly comprised relative short distances and a few include long distances, being the median of the distance 45 and 49.3 km, respectively.

**Figure 2:**
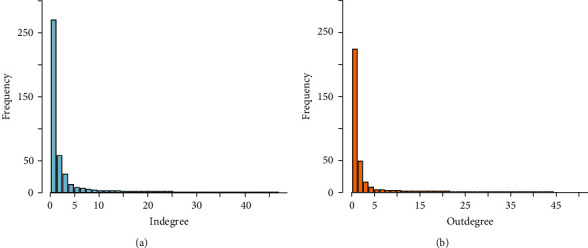
Degree distribution of premises after removing slaughterhouses. (a) Indegree; (b) outdegree. In order to improve the visualization, only frequency <1,500 were included.

We used two measures of centrality to characterize nodes that are central or important in the network: the in- and outdegree and the betweenness. A high heterogeneity in the number of contacts was found, in both indegree and outdegree, where most premises had few links and a few had a large number of connections.  Figure 2 shows the in- and outdegree distributions of premises in the GNWS. When we considered all movements in the GN, inward and outward animal movements were slightly correlated (*ρ* = 0.27 (95% CI (0.25–0.29))). After removing slaughterhouses, inward and outward movements were highly correlated (*ρ* = 0.78 (95% CI (0.77–0.80))).

Table 1 presents the betweenness, the in- and outdegree, and the number of premises reachable in two steps for the 10 premises with the highest betweenness of the GNWS and helps to identify the most influential vertices. It also shows the type of premise, either farm or livestock market, and the infection status during the initial phase of the epidemic (infected/uninfected). It can be seen that three of the 10 premises with the highest betweenness became infected during the HRP.

Other measures were estimated to assess the level of connectedness of the GNWS and are presented in a supplementary material. We found that 50% of premises can only reach one or two premises in two steps, whereas the upper 25% can reach between 22 and 119 premises (Figure S1). Additionally, the average path length, which corresponds to the mean of the lengths of the shortest paths between all pairs of vertices in the network, was 4.66. This value was compared to the average path length of 1,000 random graphs that were generated with the same number of vertices and the same density as the observed network. The average path length of the GNWS was lower than the average path length of network randomizations, suggesting that the observed network is more interconnected and may facilitate the spread of the disease between premises (Figure S2).

### 3.2. Description of the Transmission Network

**Figure 3:**
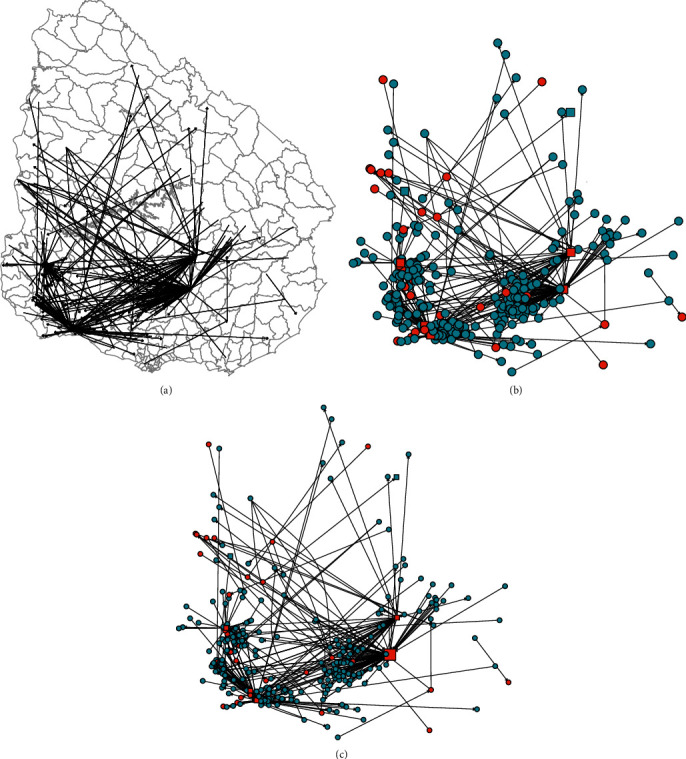
Transmission network (TN) of livestock movements during the high-risk period (HRP) of the 2001 FMDV epidemic in Uruguay (April 9, 2001 to April 27, 2001). The TN consisted of premises that were infected during the HRP and all premises connected to them by animal movements during the HRP. (a) Visualization of the network by using Uruguay´s map as the layout. (b) Farms are represented by circles, and livestock markets are represented by squares. The infectious status is shown by color; being orange, those infected premises during the HRP and light blue the uninfected ones. (c) The betweenness score determine the vertex size.

The TN consisted of premises that were infected during the HRP and all premises connected to them by animal movements between April 9, 2001 and April 27, 2001 (Figure 3). Out of the 242 farms that were infected during the HRP [6], only 34 had movements recorded during this period and are nodes of the TN. Therefore, there were no movements recorded for 208 of them. Slaughterhouses were removed as they are considered end points for FMDV transmission. Five livestock markets were considered as infected because they received animals from infected farms. The TN contained 320 nodes (313 farms and seven livestock markets) and 327 links among them (1.04 links per node).

**Figure 4:**
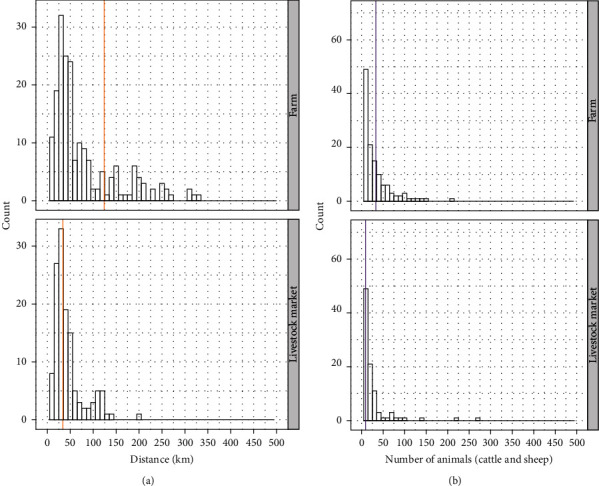
(a) Distance (km) distribution of livestock movements from farms split by the type of recipient premise (farm, livestock market) in the transmission network. (b) Distribution of the number of animals of livestock movements from farms split by the type of recipient premise of the transmission network. The vertical lines show the median of the distance and number of animals, respectively.

Most of the movements of the TN comprised cattle only (87.8%), whereas 9.2% sheep only and 3% involved both species (cattle and sheep). The 327 links among operations consisted of 133 (40.7%) from farms to livestock markets, 153 (46.8%) from livestock market to farms, and 41 between farms (12.5%). Of the total movements from farms, 76.4% were to livestock markets with a median of nine animals and a median distance of 33.7 km and 23.6% to other farms with a median of 33 animals and a median of 124 km of distance (Figure 4).

The proportion of dairy and mixed farms in the TN increased compared to the GN and the GNWS when farms were either source and recipients of animals. This increase was around 8% for dairy farms and around 14% for mixed farms when they acted as source of animals and around 12% for dairy farms and around 14% for mixed farms when they were recipient farms (Table S1). As was mentioned above, the most frequent destination of animal movements from farms was livestock markets (76.4%), with this proportion within each stratum being higher for dairy farms (85.7%) than for beef farms (75%). When it came to movements toward farms, dairy farms exclusively received animals from livestock markets, whereas the movements to beef farms consisted of 25% from other farms and 75% from livestock markets (Table S2).

**Figure 5:**
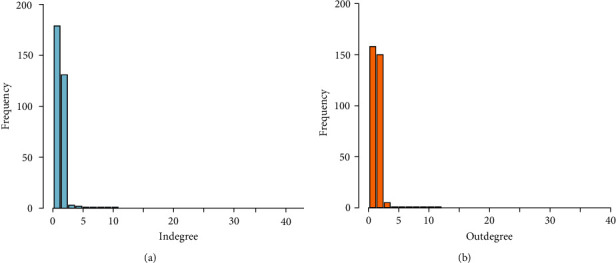
(a) Indegree distribution of premises in the transmission network; (b) outdegree distribution of premises in the transmission network.

The indegree and outdegree distributions of the TN are shown in Figure 5. The mean of the indegree and outdegree was 1.02 and the covariance was 11.41 (Table 2).

### 3.3. Estimation of the Herd Reproduction Number (R_H_)

Here, we derived the reproduction number from the average number of outcontacts of infected premises ($\overset{―}{y}\prime$) and the average probability that a contact leads to infection ($\overset{―}{q}$).

The average number of outcontacts of infected premises was 12.19 (Equation (1)).

The average probability that a contact leads to an infection ($\overset{―}{q}$) was estimated from the number of farms that were infected before the movement´s restrictions enforcement (*n* = 39 out of the 242 farms that were infected during the HRP) divided by the total number of farms included in the TN (*n* = 320). The average probability that a contact leads to an infection was 0.12. The average number of new infected farms that were caused by a typical infected farm through animal movements was *R*_H_ = 1.48 (Equation (2)).

We found that if the virus was introduced 1 week earlier, the number of infected farms involved in animal movements would be 60 (out of the 242 infected farms of the HRP), and the total number of farms included in the TN would be 411, resulting in a probability of infection 0.15. In this scenario, the average number of outcontacts of infected premises would be 14.8, and the estimated *R*_H_ would be 2.16. Considering a 3-week period instead of a 2-week period, resulted in an increase in the number of infected farms (out of the 242 farms that were infected during the HRP), which contact other farms through animal movements, leading to a higher average of outcontacts of infected premises and a higher probability that a contact leads to infection.

## 4. Discussion

The movements of infected animals played a key role in FMDV spread among premises during the HRP of the 2001 FMD epidemic in Uruguay. The similarities found between the 2001 and the current (2022) livestock networks, in terms of contact heterogeneity, underscore the importance of targeting highly connected premises, particularly livestock markets, for enhanced surveillance and implement fast control measures during epidemics.

Similar to the observed livestock movement networks in the initial phase of the 2001 FMD epidemic in the UK, the network of livestock movements during the initial phase of the 2001 FMD epidemic in Uruguay has shown a high heterogeneity of contacts among premises [15]. A right-skewed distribution of both in- and outdegree was observed, where most of the premises had few connections and a few were highly connected. This high heterogeneity of contacts among premises was a pattern that has also been observed in other networks of livestock movements [14, 17, 24], as well as in the Uruguayan network of current livestock movements. In general, premises that have many inward connections are at higher risk of being infected, while those with frequent outgoing movements have the potential to spread infectious agents widely. Therefore, it is important to identify highly connected premises in advance of FMDV introduction, so they can be targeted for surveillance and education in peacetime and for fast intervention during epidemics.

Premises that had both high in- and outdegree also tend to have high betweenness and are likely important controlling the flow from one part of the network to another part. In other words, individuals with high betweenness have an important role by connecting different parts of a network that could otherwise be less connected. Of the 10 premises with the highest betweenness of the GNWS, eight were livestock markets and two were farms. In addition, three of these premises (two livestock markets and one farm) became infected during the HRP and played a crucial role in the spread of FMDV during the 2001 FMD epidemic in Uruguay. A similar finding was reached by Ortiz-Pelaez et al. [15] in the analysis of animal movements during the initial phase of the 2001 FMD epidemic in UK. The authors found that three of the 10 premises with the highest betweenness were farms, highlighting the importance of this type of farms in the virus spread in addition to livestock markets. Intervention measures targeted to those premises with high betweenness could reduce the size of future epidemics.

The inward and outward movements were slightly correlated in the GN (Pearson's *ρ* = 0.27 (95% CI (0.25–0.29))) and after removing the slaughterhouses, this correlation substantially increased (*ρ* = 0.78 (95% CI (0.77–0.80))), indicating that those highly connected farms were not only more likely to become infected but also to spread FMDV to other farms.

In this study, we estimated FMDV transmission through livestock movements by estimating the basic reproduction number (*R*_H_). The high covariance between incoming and outgoing movements may explain that *R*_H_ was above one, confirming the high-risk animal movements played in sustained transmission of FMDV during the epidemic in Uruguay. Previous studies have evaluated the potential for transmission for an infection that may spread through the contact network by looking at the contribution of covariance between contact rates [25, 26]. In this sense, we estimated the mean rates of contacts and the covariance between them for the network of livestock movements after removing livestock markets either as source or as recipients to illustrate the key role that livestock market played in spreading FMDV during the initial phase of the epidemic. As expected, when we removed livestock markets, the magnitude of the covariance between contact rates resulted in a sharp decrease of *R*_H_ from 1.48 to 0.03 (Table 2). We also estimated the contact rates and covariance between them for the network of the current livestock movements in Uruguay (2022). No significant differences were found in terms of the mean contact rates and the covariance between the networks of 2001 and 2022. This suggests that if FMDV is introduced to Uruguay, finds its way to a livestock market and is not detected early, several farms may be already infected only through animal movements by the time the first outbreak is detected leading to a large epidemic as that in 2001.

While the majority of the movements (95%) occurred over a range of 37–129 km, 5% of them happened at 285–490 km. These long-distance movements may explain the large geographical spread of FMDV throughout the country during the HRP [6]. Most of the farms involved in animal movements were beef farms (around 90%) and more than half were large farms (>463 animals). However, we observed that the proportion of dairy and mixed farms was higher in the TN compared to its proportions in the GN and the GNWS. This shows that dairy farms contributed to FMDV transmission through the movement of animals during the initial phase of this epidemic. This contribution may be mainly explained by the fact that dairy farms carried out more animal movements through livestock markets compared to beef farms in the TN.

We acknowledge some of the limitations of this study, which fall into two categories: related to the dataset and assumptions made for the analyses. Regarding the first, some omissions may exist in the movement dataset due to lack of movement declaration by livestock owners. These omissions may occur with very low frequency and usually comprise neighbors' farms trading, which likely involve few animals. Although these omissions, our results provide an overall structure of the livestock movement networks and its implication in the FMDV transmission during the initial phase of the 2001 epidemic in Uruguay. In addition, uncertainty around the date of infection of the herd may also exist. For this reason, we also estimated the basic reproduction number assuming that the virus was introduced 1 week earlier. In this case, *R*_H_ was higher than our estimation (2.16 vs. 1.48), which means that our assumed time of introduction results in a more conservative assessment of the contribution of livestock movements to FMDV spread during this epidemic. More confidence in the estimation of the time of infection would allow us to estimate a more precise probability of transmission from pairs of contacts and, therefore, estimate a more accurate *R*_H_. Regarding the assumptions made, we assumed that premises included in the TN were infected via livestock movements during the HRP, omitting other links between farms where infection can spread such as shared employees and equipment, the milk collection and feed transport vehicles, and veterinarian's visits and neighboring.

Our study can contribute to a better understanding of the role of livestock movements in the spread of FMDV during the initial phase of the 2001 FMD epidemic in Uruguay when the virus was yet undetected. However, many farms that were infected during this period had not livestock movements recorded, so the movements of animals did not explain the whole transmission. This highlights the importance of other routes of transmission that involve contact links among premises that are hard to record and reflects the need of using models able to capture it to analyze FMDV transmission between premises. Further research is planned in order to understand the overall transmission during this epidemic.

## 5. Conclusions

In conclusion, the movements of infected animals during the HRP of the 2001 FMD epidemic in Uruguay had an important contribution to the virus spread among premises. Livestock markets and highly connected farms were responsible for the early long-distance spread of FMDV before a ban on animal movements had been implemented. The analysis of the 2022 network shows that this network is similar to that of 2001 and highlights the importance of targeting highly connected premises, particularly livestock markets, for surveillance, target early detection, and implement interventions during epidemics.

## Figures and Tables

**Table 1:** Connectedness metrics of the 10 premises with the highest betweenness of the network without slaughterhouses.

S. No.	Type	Betweenness	Indegree	Outdegree	Number of premises reachable in two steps	Infection status
1	Livestock market	40,575	60	42	58	Uninfected
2	Livestock market	36,569	48	37	47	Infected
3	Livestock market	29,617	34	30	42	Uninfected
4	Farm	22,919	1	1	41	Infected
5	Livestock market	22,859	1	39	48	Infected
6	Livestock market	22,832	148	77	86	Uninfected
7	Livestock market	20,966	35	15	18	Uninfected
8	Livestock market	20,003	22	20	25	Uninfected
9	Farm	19,249	1	1	44	Uninfected
10	Livestock market	18,902.5	21	23	29	Uninfected

Type refers to the premise type, whether it is a farm or a livestock market, and the infection status indicates if the premise was infected or not during the high-risk-period.

**Table 2:** Mean of in- and outdegree and covariance between them for networks of livestock movements in 2001 and current livestock movements (2022).

Networks	Mean indegree ($\overset{―}{x}$)	Mean outdegree ($\overset{―}{y}$)	*cov*(*x*, *y*)	*R*_H_
2001				
General	1.18	1.18	7.50	0.91
General WS^a^	0.98	0.98	9.52	1.28
Transmission	1.02	1.02	11.41	1.48
Transmission WLM^b^	0.68	0.68	−0.31	0.03
2022				
General	1.23	1.23	7.06	0.84
General WS^a^	1.07	1.07	8.8	1.11
General WLM^b^	0.88	0.88	−0.09	0.09

^a^WS: without slaughterhouses. ^b^WLM: without livestock markets. General network 2001 includes all livestock movements in the period between April 9, 2001 and April 27, 2001, which was the high-risk period (HRP) during the 2001 FMD epidemic in Uruguay. The same period of the year was considerate to define the general network in 2022. Networks without slaughterhouses did not include movements to slaughterhouses. The transmission network 2001 consisted of premises that were infected during the HRP and all premises connected to them by animal movements during the HRP. The transmission network 2001 without livestock markets did not include the movements that involve livestock markets as source or recipients. The *R*_H_ was estimated for all networks by using Equation (2) and assuming the same probability of infection as that in the transmission network (*p*=0.12).

## Data Availability

The data used to support the findings of this study were supplied by the Official Veterinary Services (Dirección General de los Servicios Ganaderos (DGSG)) of the Ministry of Livestock, Agriculture and Fisheries of Uruguay (Ministerio de Ganadería, Agricultura y Pesca (MGAP)). It is confidential as it contains private information of livestock owners of Uruguay and so cannot be made freely available. Requests for access to these data should be made to the DGSG, MGAP, Uruguay, dgsg@mgap.gub.uy.

## Appendix

Each premise has a certain number of contacts *k* (0, 1, 2, 3 …), each number *k* with probability distribution *P*_*k*_.

The average number of contacts is, thus, by definition $\overset{―}{k}=\sum_{k=0}^{\infty }\;kP_{k}$, and the variance is $var\;k=\sum_{k=0}^{\infty }{\left(k-\overset{―}{k}\right)}^{2}\;P_{k}$.

Infected premises have more contacts than other premises, i.e., *k*′ number of contacts, with distribution $P_{k^{\prime }}=\frac{kP_{k}}{\overset{―}{k}}$. This is the (in)famous question why your friends on a social network site will have more friends than the average person on that site (so typical on average more than you).

For one thing, when *k*=0, *P*_*k*′_=0, and premises cannot get infected.

If $k=1,P_{k^{\prime }}=\frac{P_{1}}{\overset{―}{k}}$, and premises can get infected but they never will infect other premises.

The average number of contacts of an infected premise is $\overset{―}{k^{\prime }}=\sum_{k=0}^{\infty }\;k\frac{kP_{k}}{\overset{―}{k}}=\sum_{k=0}^{\infty }\frac{k^{2}P_{k}}{\overset{―}{k}}$.

Note: $var\;k=\sum_{k=0}^{\infty }\;k^{2}P_{k}-{\left(\sum_{k=0}^{\infty }k\;P_{k}\right)}^{2}$.

The average number of contacts of an infected premise can be expressed as follows:

(3) $$\begin{array}{c} \overset{―}{k^{\prime }}=\frac{var\;k}{\overset{―}{k}}+\overset{―}{k}. \end{array}$$

As one contact is needed for infection, so the average number of contacts of an infected premise is $\overset{―}{k^{\prime }}=\frac{var\;k}{\overset{―}{k}}+\overset{―}{k}-1$.

*x* is the indegree with distribution *P*_*x*_ and *y* is the outdegree with distribution *P*_*y*_. For each premise with *x* indegree and *y* oudegree, the probability is *P*_*x*,*y*_.

For infected premises, the average number of outcontacts is given by:

(4) $$\begin{array}{c} \overset{―}{y}=\sum_{x=0}^{\infty }\sum_{y=0}^{\infty }\;y\frac{x\;P_{x,y}}{\overset{―}{x}}. \end{array}$$

*Note*: $\text{cov}\left(x,y\right)=\sum_{x=0}^{\infty }\;\sum_{y=0}^{\infty }\;xyPx,y-\sum_{x=0}^{\infty }\;\sum_{y=0}^{\infty }\;xP_{x}\sum_{y=0}^{\infty }\;\sum_{x=0}^{\infty }yP_{y}$.

The average number of outcontacts of infected premises can be expressed as (because we do not need to account for the one incoming contact in the number of outgoing contacts):

(5) $$\begin{array}{c} \overset{―}{y}\prime =\frac{\text{cov}\left(x,y\right)}{\overset{―}{x}}+\overset{―}{y}. \end{array}$$

## Supplementary Materials

Supplementary 1 Table S1: number of animal movements and proportion expressed as percentage of farms of different production purposes (beef, dairy, and mixed (beef and dairy)) and different herd sizes (<36, 36–134, 136–463, >463), either as source and recipient of animals, for livestock networks of the initial phase of the 2001 epidemic in Uruguay.

Supplementary 2 Figure S1: number of premises reachable in *k* ≤ 2 steps for all premises included in the general network without slaughterhouses. Figure S2: average path length of 1,000 randomly generated networks with the same number of vertices and density as the general network without slaughterhouses (GNWS) and the average path length for the observed network. Table S2: number of animal movements and proportion expressed as percentage of farms of different production purposes (beef, dairy, and mixed (beef and dairy)) by destination of movements when farms were source, and by origin when farm were recipients, in the livestock networks of the initial phase of the 2001 epidemic in Uruguay. A text file with the *R* code was used for the analyses.
