# Supplementary material for: Animal Movements and FMDV Transmission during the High-Risk Period of the 2001 FMD Epidemic in Uruguay
Source: Transbound Emerg Dis. 2023 Nov 11;2023:8883502. doi: 10.1155/2023/8883502 (PMC12016683; doi:10.1155/2023/8883502)
Supplement: Supplementary 2 — Figure S1: number of premises reachable in k ≤ 2 steps for all premises included in the general network without slaughterhouses. Figure S2: average path length of 1,000 randomly generated networks with the same number of vertices and density as the general network without slaughterhouses (GNWS) and the average path length for the observed network. Table S2: number of animal movements and proportion expressed as percentage of farms of different production purposes (beef, dairy, and mixed (beef and dairy)) by destination of movements when farms were source, and by origin when farm were recipients, in the livestock networks of the initial phase of the 2001 epidemic in Uruguay. A text file with the R code was used for the analyses. [file 8883502.f2.docx]

Table S 1 Table S1 Number of animal movements and proportion expressed as percentage, of farms of different production purpose (beef, dairy and mixed (beef and dairy) by destination of movements when farms were source, and by origin when farm were recipients, in the livestock networks of the initial phase of the 2001 epidemic in Uruguay. y. GN=General Network. GNWS=General Network Without Slaughterhouses. TN= Transmission Network. The GN includes all livestock movements in the period between 09-04 and 27-04 in 2001. The GNWS did not include movements to slaughterhouses. The Transmission Network 2001 included all links from or to farms that were infected during the high-risk period.

|  | **Source** | | | |  | **Recipient** | | | |  |
| --- | --- | --- | --- | --- | --- | --- | --- | --- | --- | --- |
| **Production type** | **Beef** | **Dairy** | **Mixed** |  |  | **Beef** | **Dairy** | **Mixed** |  |  |
|  | **n (%)** | **n (%)** | **n (%)** |  |  | **n (%)** | **n (%)** | **n (%)** |  |  |
| GN | 4983(89) | 308 (5.5) | 308 (5.5) |  |  | 2352(90.6) | 126(4.6) | 117(4.5) |  |  |
| GNWS | 2496(89.8) | 136(4.9) | 146(5.3) |  |  | 2352(90.6) | 126(4.6) | 117(4.5) |  |  |
| TN | 112 (68.3) | 21(12.8) | 31(18.9) |  |  | 115(64.6) | 30(16.9) | 33(18.5) |  |  |
| **Farm size** | **<36** | **36-134** | **134-463** | **>463** |  | **<36** | **36-134** | **134-463** | **>463** |  |
|  | **n (%)** | **n (%)** | **n (%)** | **n (%)** |  | **n (%)** | **n (%)** | **n (%)** | **n (%)** |  |
| GN | 294(5.2) | 728(12.9) | 1446(25.7) | 3168(56.2) |  | 242(9.2) | 440(16.7) | 642(24.4) | 1309(49.7) |  |
| GNWS | 166(5.9) | 418(14.9) | 862(30.8) | 1357(48.4) |  | 242(9.2) | 440(16.7) | 641(24.4) | 1308(49.7) |  |
| TN | 14(8.5) | 43(26) | 43(26) | 65(39.5) |  | 22(12.1) | 42(23.1) | 42(23.1) | 76(41.8) |  |
|  | | | | | | | | | |  |
|  |  |  |  |  |  |  |  |  |  |  |


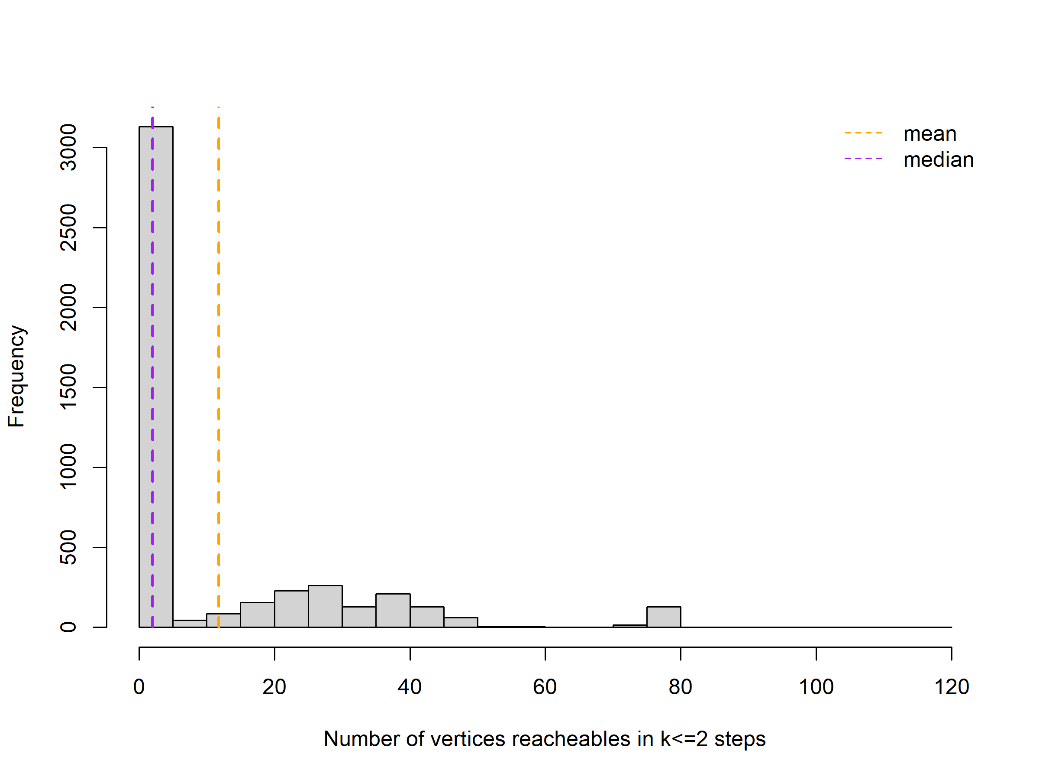


Figure S1 Number of premises reachable in k<=2 steps for all premises included in the network after removing slaughterhouses (GNWS). The mean (12 premises) is shown in orange, while the median (2 premises) in purple


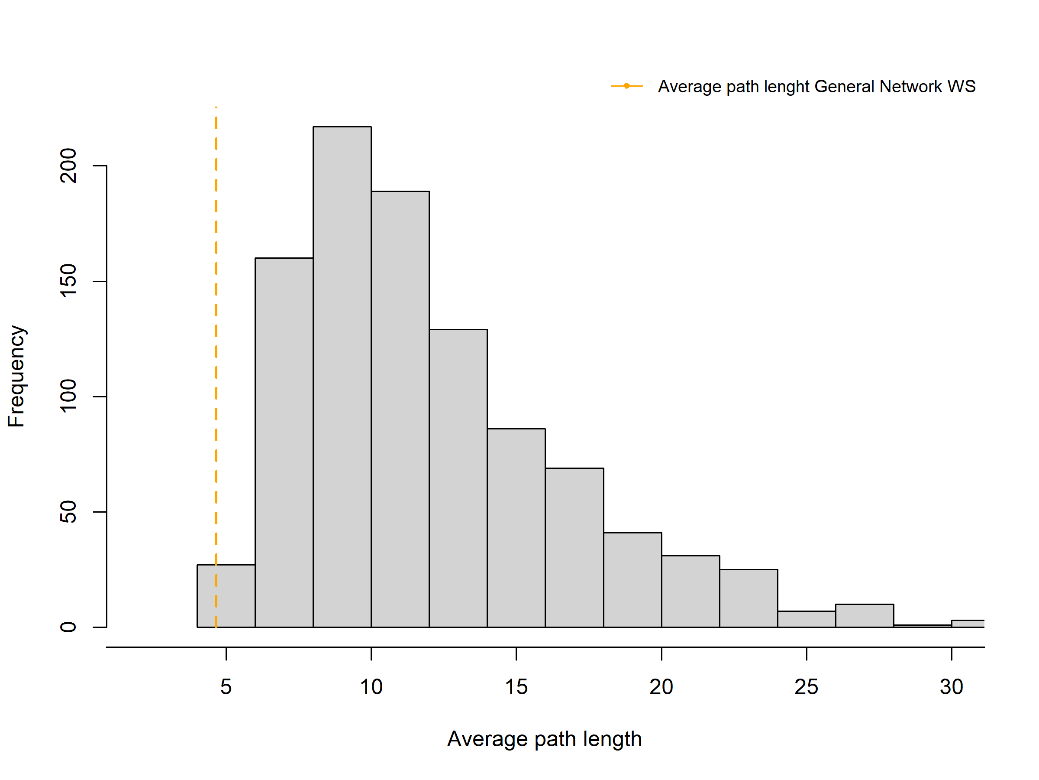


Figure S2 Average path length of 1000 randomly generated networks with the same number of vertices and density as the General Network without Slaughterhouses (GNWS) and the average path length for the observed network (orange vertical line).

Table S 2 Number of animal movements, and proportion expressed as percentage, of farms of different production purpose (beef, dairy and mixed (beef and dairy) by destination of movements when farms were source, and origin when farm were recipients, in the livestock networks of the initial phase of the 2001 epidemic in Uruguay. GN=General Network. GNWS=General Network Without Slaughterhouses. TN= Transmission Network. The GN includes all livestock movements in the period between 09-04 and 27-04 in 2001. The GNWS did not include movements to slaughterhouses. The Transmission Network 2001 included all links from or to farms that were infected during the high-risk period.

|  | ***Farm as source of animals*** | | | | | |  | ***Farm as recipient of animals*** | | | | | |  |
| --- | --- | --- | --- | --- | --- | --- | --- | --- | --- | --- | --- | --- | --- | --- |
|  |  | **GN** | | **GNWS** | | **TN** | | **GN** | | **GNWS** | | **TN** | |  |
|  |  | **n** | **%** | **n** | **%** | **n** | **%** | **n** | **%** | **n** | **%** | **n** | **%** |  |
| **beef** | farm | 1189 | 23.9 | 1189 | 47.6 | 28 | 25 | 1249 | 53.1 | 1249 | 53.1 | 28 | 24.3 |  |
|  | livestock market | 1307 | 26.2 | 1307 | 52.4 | 84 | 75 | 1103 | 46.9 | 1103 | 46.9 | 87 | 75.7 |  |
|  | slaughterhouse | 2487 | 49.9 |  |  |  |  |  |  |  |  |  |  |  |
|  |  | 4983 |  | 2496 |  | 112 |  | 2352 |  | 2352 |  | 115 |  |  |
|  |  |  |  |  |  |  |  |  |  |  |  |  |  |  |
| **dairy** | farm | 69 | 22.4 | 69 | 50.7 | 3 | 14.3 | 40 | 31.7 | 40 | 31.7 | 0 |  |  |
|  | livestock market | 67 | 21.8 | 67 | 49.3 | 18 | 85.7 | 86 | 68.3 | 86 | 68.3 | 30 | 100 |  |
|  | slaughterhouse | 172 | 55.8 |  |  |  |  |  |  |  |  |  |  |  |
|  |  | 308 |  | 136 |  | 21 |  | 126 |  | 126 |  | 30 |  |  |
|  |  |  |  |  |  |  |  |  |  |  |  |  |  |  |
| **mixed** | farm | 52 | 16.9 | 52 | 35.6 | 6 | 19.4 | 44 | 37.6 | 44 | 37.6 | 8 | 24.2 |  |
|  | livestock market | 94 | 30.5 | 94 | 64.4 | 25 | 80.6 | 73 | 62.4 | 73 | 62.4 | 25 | 75.8 |  |
|  | slaughterhouse | 162 | 52.6 |  |  |  |  |  |  |  |  |  |  |  |
|  |  | 308 |  | 146 |  | 31 |  | 117 |  | 117 |  | 33 |  |  |
|  | | | | | | | | | | | | | |  |
|  |  |  |  |  |  |  |  |  |  |  |  |  |  |  |
